# Hemorrhagic fever with renal syndrome complicated by reversible secondary hyperparathyroidism during renal recovery: a case report

**DOI:** 10.3389/fmed.2026.1811696

**Published:** 2026-05-08

**Authors:** Haixia Hu, Xiuxian Zang, Zuolong Liu, He Yan, Bing Lv

**Affiliations:** Department of Emergency, The First Hospital of Jilin University, Changchun, Jilin, China

**Keywords:** acute kidney injury, case report, hemorrhagic fever with renal syndrome, hypocalcemia, secondary hyperparathyroidism

## Abstract

Hemorrhagic fever with renal syndrome (HFRS) is a hantavirus-induced zoonosis characterized by fever, hemorrhagic manifestations, and acute kidney injury (AKI). Endocrine sequelae have been increasingly recognized after HFRS, yet parathyroid dysfunction has rarely been reported. Secondary hyperparathyroidism (SHPT) is classically associated with chronic kidney disease, but severe AKI may also disrupt mineral metabolism and trigger abnormal parathyroid hormone (PTH) responses. In this study, we report a case of secondary hyperparathyroidism suspected to be triggered by hemorrhagic fever with renal syndrome, a presentation that has been rarely described in the literature. In addition, we provide a review of the existing evidence regarding endocrine involvement in HFRS.

## Introduction

Hemorrhagic fever with renal syndrome (HFRS) is an acute rodent-borne zoonotic disease caused by hantaviruses and remains endemic in parts of Asia and Europe (1). It is clinically characterized by high fever, hemorrhagic manifestations, thrombocytopenia, and acute kidney injury (AKI), with severe cases typically progressing through febrile, hypotensive, oliguric, diuretic, and convalescent phases. Renal involvement represents the hallmark of HFRS and is primarily attributed to virus-induced endothelial dysfunction, increased capillary permeability, immune-mediated injury, and microvascular damage (2). Beyond renal and hematologic abnormalities, HFRS is increasingly recognized as a multisystem disorder. Endocrine complications, including hypopituitarism, thyroid dysfunction, and gonadal involvement, have been reported during both the acute phase and long-term follow-up, indicating that endocrine axes may be particularly vulnerable to the systemic inflammatory and hemodynamic disturbances associated with hantavirus infection (3).

Secondary hyperparathyroidism (SHPT) is most commonly associated with chronic kidney disease and arises from disrupted calcium-phosphate and vitamin D homeostasis, leading to sustained parathyroid hormone (PTH) overproduction. Although traditionally regarded as a chronic complication, profound alterations in mineral metabolism may also occur in the setting of severe AKI (4). In HFRS, rapid and dynamic changes in renal function, together with immune activation and endothelial injury, may create conditions conducive to dysregulated PTH secretion. To our knowledge, no prior case of reversible SHPT during the recovery phase of HFRS-associated AKI has been reported. Here, we report a unique case of HFRS complicated by reversible secondary hyperparathyroidism in a previously healthy young man, aiming to expand the recognized spectrum of endocrine complications of HFRS and to raise clinical awareness of calcium-phosphate disturbances during the recovery phase of the disease. Written informed consent was obtained from the patient for publication of this case report.

## Case presentation

A 34-year-old male forestry police officer with no significant past medical history presented with 5 days of fever and lumbar pain and 2 days of oliguria. He was admitted to the First Hospital of Jilin University on 6 October 2024. Physical examination revealed a temperature of 38 °C, blood pressure of 88/52 mmHg and pulse of 98 beats/min. The patient appeared flushed with conjunctival injection, and bilateral costovertebral angle tenderness was elicited. Urine output over the preceding 24 h was 400 mL.

Initial laboratory studies demonstrated marked leukocytosis (35.75 × 10^9^/L, reference range 3.5–9.5 × 10^9^/L) and thrombocytopenia (54 × 10^9^/L, reference range 100–350 × 10^9^/L). Urinalysis showed heavy proteinuria (+++). Serum creatinine was 397 μmol/L (reference range 57–97 μmol/L) and blood urea nitrogen 18.08 mmol/L (reference range 3.1–8.0 mmol/L). Serum calcium was 2.38 mmol/L (reference range 2.20–2.65 mmol/L). HFRS-specific immunoglobulin M was positive. Abdominal ultrasonography revealed bilaterally enlarged kidneys with diffuse parenchymal hyperechogenicity and mild perirenal effusion. Based on these findings, a diagnosis of HFRS was established, and supportive care together with antiviral therapy was initiated. Despite treatment, serum creatinine and urea nitrogen increased further and serum calcium declined. On hospital day 5, continuous renal replacement therapy (CRRT) was commenced for 24 h. By day 7, urine output exceeded 500 mL/day and serum creatinine began to fall, signifying the onset of the diuretic phase. Serum calcium concurrently rose toward normal.

**Figure 1:**
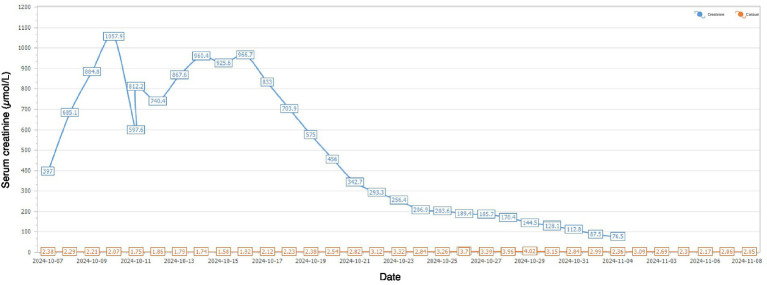
Dynamic changes in serum creatinine levels during hospitalization from October 7 to November 8, 2024.

**Figure 2:**
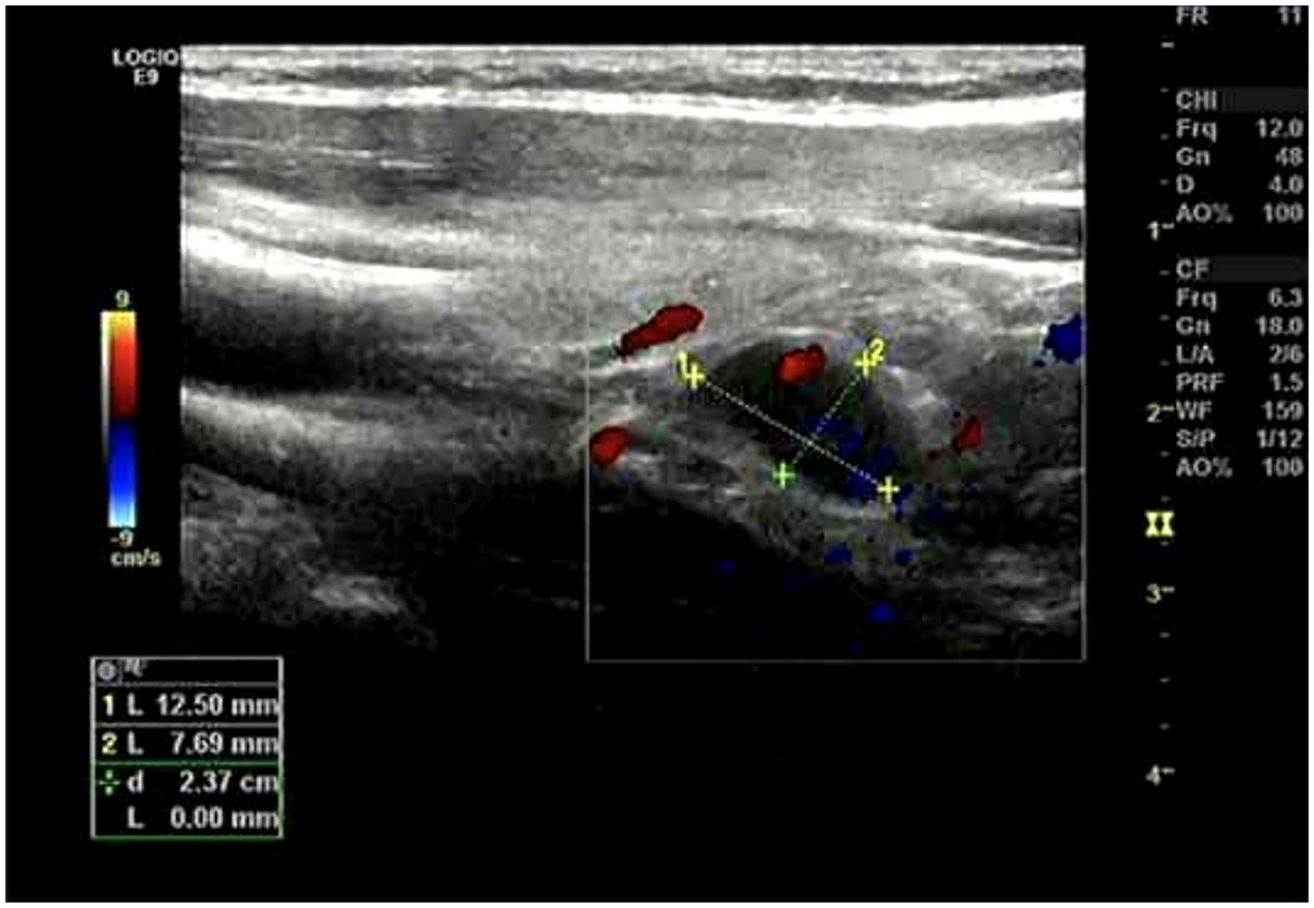
Ultrasound examination demonstrated a hypoechoic nodule located posterior–inferior to the right thyroid lobe, suggestive of enlarged and functionally active parathyroid tissue.

**Figure 3:**
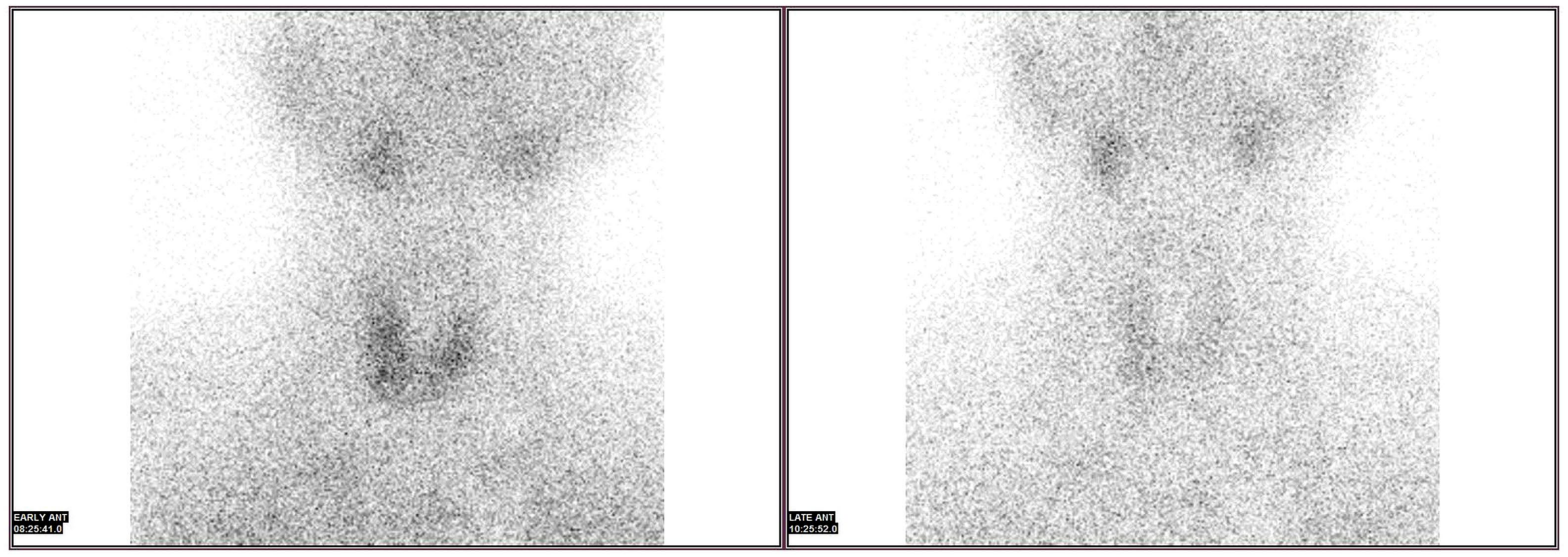
SPECT/CT showed a focal area of increased radiotracer uptake at the inferior pole of the right thyroid lobe, suggestive of a hyperfunctioning parathyroid gland.

**Figure 4:**
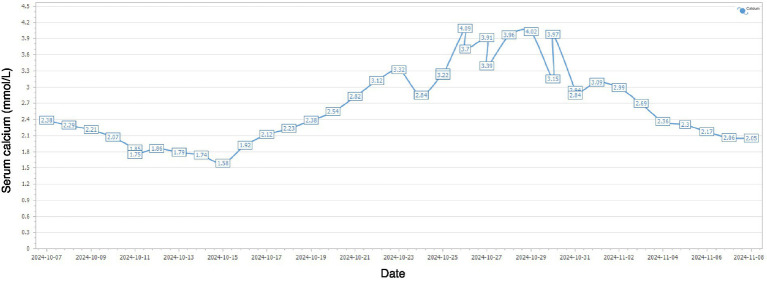
Dynamic changes in serum calcium levels during hospitalization from October 7 to November 8, 2024.

After 14 days of therapy, creatinine levels continued to decrease (Figure 1) and daily urine output exceeded 5 L; however, serum calcium rose above normal, peaking at 4.09 mmol/L. PTH was markedly elevated at 618.4 pg/mL (normal 15–68.3 pg/mL), suggesting hyperparathyroidism. Serum alkaline phosphatase (ALP) was 69.4 U/L at admission (October 6, 2024) and increased to 111.5 U/L approximately 3 weeks later (October 29, 2024) (reference range, 45–125 U/L). Neck ultrasonography demonstrated a hypoechoic nodule located posterior-inferior to the right thyroid lobe (Figure 2), and parathyroid SPECT/CT imaging showed a focal area of increased radiotracer uptake at the corresponding site (Figure 3). These findings supported the diagnosis of SHPT. The patient was treated with aggressive intravenous hydration, a loop diuretic, subcutaneous calcitonin and intravenous zoledronic acid. Over 9 days, serum calcium returned to normal levels. No supplemental calcium or active vitamin D preparations were administered during the hypocalcemic phase. He was discharged 1 month after admission. At a three-month follow-up, PTH had decreased to 59.4 pg/mL (reference range 15–65 pg/mL) and serum calcium remained within the reference range (Figure 4).

## Discussion

HFRS and SHPT have both been extensively investigated as independent clinical entities; however, reports describing their coexistence are exceedingly rare. To date, no systematic studies or clinical guidelines have addressed hyperparathyroidism occurring in the context of HFRS, and the available literature is limited to isolated case observations. This lack of evidence poses significant challenges for clinicians when encountering such complex presentations, often resulting in delayed recognition and suboptimal management. In this study, we report a case of hemorrhagic fever with renal syndrome complicated by secondary hyperparathyroidism. Analysis of the potential pathophysiological mechanisms suggests that a causal relationship between these two conditions cannot be excluded.

Beyond its characteristic renal and hematologic involvement, HFRS is increasingly recognized as a multisystem disorder with potential endocrine sequelae. A retrospective study reported that endocrine abnormalities occurred in approximately 18% of patients within 2 years following HFRS, underscoring the vulnerability of endocrine axes during and after infection (3). Among these, hypopituitarism represents the most frequently reported complication (5, 6). Several mechanisms have been proposed, including hantavirus-induced thrombocytopenia (7, 8) and endothelial dysfunction leading to pituitary hemorrhage (9), ischemic injury secondary to hypotension and vasospasm during the acute phase, direct viral invasion of pituitary tissue (8), and virus-triggered autoimmune hypophysitis (10, 11). Long-term follow-up studies have demonstrated mild pituitary atrophy on magnetic resonance imaging in most affected individuals, with a minority requiring lifelong hormone replacement therapy (12).

Thyroid dysfunction has also been reported in association with HFRS. The prevalence of primary hypothyroidism in HFRS survivors is estimated to be nearly threefold higher than that in age-matched populations (13). Central or mixed hypothyroidism may occur in rare cases, possibly mediated by cytokine-driven endocrine cell injury or molecular mimicry between viral antigens and thyroid tissue, resulting in autoimmune thyroid disease (11). In addition, gonadal involvement has been documented, including cases of orchitis attributed to vascular dysfunction and increased capillary permeability (14, 15). Previous studies have shown that endocrine disturbances are common during nephropathia epidemica, with approximately 56% of patients exhibiting abnormalities in the gonadal and/or thyroid axis during the acute phase, and 17% developing chronic, overt hormonal deficiencies after a median follow-up of 5 years (16).

In contrast to these relatively well-documented endocrine complications, SHPT associated with HFRS is exceedingly uncommon. Traditionally, SHPT is regarded as a chronic complication of chronic kidney disease (CKD), resulting from persistent disturbances in calcium-phosphate and vitamin D homeostasis. However, severe AKI can also induce acute, reversible secondary hyperparathyroidism independent of CKD. Acute impairment of renal function leads to rapid phosphate retention, hypocalcemia, and reduced activation of vitamin D, which collectively stimulate PTH secretion. Moreover, systemic inflammation and uremic toxicity may downregulate calcium-sensing receptor (CaSR) and vitamin D receptor (VDR) expression in parathyroid tissue, resulting in inappropriately elevated PTH levels and transient functional activation of the parathyroid glands, with possible reversible hypertrophy-like changes under sustained stimulation. This pathophysiological pattern is consistent with the clinical course observed in our patient.

In the present case, serum calcium exhibited a dynamic biphasic pattern closely associated with renal function. During the first week of hospitalization, progressive AKI was accompanied by declining serum calcium levels, consistent with impaired phosphate excretion, hyperphosphatemia, and reduced renal synthesis of 1,25-dihydroxyvitamin D. As renal function began to recover during the diuretic phase, serum calcium initially normalized but subsequently rose paradoxically to markedly supraphysiological levels, persisting for nearly 2 weeks despite continued improvement in renal indices. Of note, PTH levels remained markedly elevated despite the development of hypercalcemia, indicating a transient loss of normal feedback regulation rather than true autonomous secretion. Under physiological conditions, PTH is rapidly suppressed by hypercalcemia because of its short half-life. The failure of PTH to decline in this patient suggested transient functional dysregulation of the parathyroid glands induced by prolonged stimulation during severe AKI, a mechanism supported by previous studies demonstrating that severe and prolonged AKI may lead to sustained parathyroid stimulation and even hypertrophy (17).

Notably, primary parathyroid adenoma was highly unlikely in this patient. Primary hyperparathyroidism typically presents with spontaneous hypercalcemia, persistently unsuppressed PTH, and normal renal function, whereas this patient initially developed hypocalcemia with compensatory PTH elevation during AKI. At the 3-month follow-up, both serum calcium and PTH returned to stable normal ranges, which is inconsistent with a primary adenoma. The parathyroid nodule identified on ultrasonography and SPECT/CT more likely represented reactive functional activation with possible reversible hypertrophy-like changes secondary to prolonged metabolic stimulation. No exogenous calcium or active vitamin D supplementation was administered during the hypocalcemic phase. ALP levels remained within normal limits throughout the clinical course, indicating no excessive bone turnover.

Neck ultrasonography revealed a hypoechoic nodule located posteroinferior to the right thyroid lobe, and parathyroid SPECT/CT imaging demonstrated a focal area of increased radiotracer uptake at the corresponding site. It should be clarified that imaging findings alone cannot distinguish primary from secondary hyperparathyroidism. However, in the clinical context of HFRS-induced severe AKI, these imaging findings strongly support the diagnosis of SHPT. The enlarged and functionally overactive parathyroid tissue represented functionally active parathyroid tissue under sustained stimulation, possibly associated with reversible hypertrophy-like changes rather than definitive structural hyperplasia. The diagnosis of SHPT was further supported by the complete reversal of biochemical abnormalities after recovery of renal function.

Based on an analysis of the underlying pathophysiological mechanisms, a potential causal relationship between the two conditions cannot be excluded. First, AKI is a hallmark manifestation of HFRS and is frequently accompanied by disturbances in calcium-phosphate homeostasis. Reduced glomerular filtration results in phosphate retention, promoting calcium-phosphate precipitation in soft tissues and further lowering serum calcium concentrations. At the same time, impaired renal activation of vitamin D diminishes intestinal calcium absorption and removes negative feedback on PTH secretion, collectively promoting sustained parathyroid stimulation and the development of SHPT, which in severe cases may be associated with adaptive hypertrophy as suggested in previous literature (18, 19). The persistent hypocalcemia observed early in our patient’s clinical course is consistent with this mechanism.

Second, dysregulation of calcium-sensing receptor (CaSR) and vitamin D receptor (VDR) signaling may also contribute to this process. CaSR plays a crucial role in maintaining calcium homeostasis by regulating PTH secretion, parathyroid cell proliferation, and renal calcium handling. Active vitamin D suppresses PTH synthesis through VDR-mediated transcriptional regulation. Severe renal impairment in HFRS reduces vitamin D activation and may downregulate VDR expression in parathyroid tissue, thereby weakening 1,25-dihydroxyvitamin D-dependent gene regulation and facilitating excessive PTH production (20).

Finally, virus-induced immune dysregulation may represent an additional pathogenic pathway. Secondary hyperparathyroidism has been reported in association with several viral infections, including hepatitis B virus, hepatitis C virus (21, 22), SARS-CoV-2 (23), and human cytomegalovirus (24). In these conditions, immune activation, autoantibody formation, or alterations in the calcium “set-point” have been implicated in uncontrolled PTH synthesis and secretion. Given the established capacity of hantavirus infection to trigger immune-mediated endocrine disorders, it is plausible that similar mechanisms may contribute to parathyroid hyperactivity in rare cases, although direct evidence remains lacking.

Taken together, this case may expand the spectrum of endocrine complications associated with HFRS and suggests that SHPT, although rare, may occur during the recovery phase of the disease. Careful monitoring of calcium metabolism and parathyroid function should therefore be considered in patients with severe renal involvement or unexplained hypercalcemia following HFRS. Further clinical and mechanistic studies are warranted to elucidate the causal relationship and underlying pathways linking hantavirus infection and parathyroid dysfunction.

This report has several limitations. First, repeat imaging of the parathyroid nodule was not performed during follow-up, and therefore its radiologic evolution could not be assessed. Given the complete normalization of serum calcium and PTH after recovery of renal function, the nodule was more likely related to transient functional activation with possible reversible hypertrophy-like changes, rather than confirmed structural hyperplasia, as no histological or longitudinal imaging evidence was available. Second, 24-h urinary calcium was not monitored during either the treatment period or follow-up, which limited a more comprehensive evaluation of dynamic changes in calcium metabolism throughout the disease course. Third, the diagnosis of secondary hyperparathyroidism was based on the integrated assessment of clinical background, biochemical findings, imaging features, and biochemical recovery during follow-up rather than pathological confirmation. Nevertheless, the normalization of serum calcium and PTH after recovery of renal function strongly supported a reversible process rather than persistent autonomous parathyroid disease. Furthermore, current evidence regarding parathyroid structural changes in AKI is limited, and the interpretation in this case should be considered hypothesis-generating rather than definitive.

## Conclusion

This case highlights a rare endocrine complication associated with HFRS. Although direct evidence of causality is lacking, SHPT may develop during renal recovery in the setting of severe HFRS-related AKI, potentially as a consequence of dynamic disturbances in calcium-phosphate and vitamin D homeostasis. Clinicians should be aware of this possible association and consider monitoring mineral metabolism and parathyroid function in patients with severe renal involvement or unexplained hypercalcemia during the convalescent phase of HFRS.

## Data Availability

The original contributions presented in the study are included in the article/supplementary material, further inquiries can be directed to the corresponding author.
